# TMCO1 is upregulated in breast cancer and regulates the response to pro-apoptotic agents in breast cancer cells

**DOI:** 10.1038/s41420-024-02183-0

**Published:** 2024-10-01

**Authors:** Alice H. L. Bong, Mélanie Robitaille, Sichun Lin, Amy McCart-Reed, Michael Milevskiy, Stéphane Angers, Sarah J. Roberts-Thomson, Gregory R. Monteith

**Affiliations:** 1 School of Pharmacy, The University of Queensland, Woolloongabba, QLD Australia; 2 Donelly Centre, University of Toronto, Toronto, ON M5S 1A8 Canada; 3 UQ Centre for Clinical Research, Faculty of Medicine, The University of Queensland, Herston, QLD Australia; 4 ACRF Cancer Biology and Stem Cells, The Walter and Eliza Hall Institute of Medical Research, Parkville, VIC Australia; 5 Department of Medical Biology, The University of Melbourne, Parkville, Australia; 6 Leslie Dan Faculty of Pharmacy, University of Toronto, Toronto, ON M5S 1A2 Canada; 7 Department of Biochemistry, Temerty Faculty of Medicine, University of Toronto, Toronto, ON M5S 1A8 Canada

**Keywords:** Cell death, Target identification, Calcium signalling

## Abstract

The release of Ca^2+^ ions from endoplasmic reticulum calcium stores is a key event in a variety of cellular processes, including gene transcription, migration and proliferation. This release of Ca^2+^ often occurs through inositol 1,4,5-triphosphate receptors and the activity of these channels and the levels of stored Ca^2+^ in the endoplasmic reticulum are important regulators of cell death in cancer cells. A recently identified Ca^2+^ channel of the endoplasmic reticulum is transmembrane and coiled-coil domains 1 (TMCO1). In this study, we link the overexpression of *TMCO1* with prognosis in node-positive basal breast cancer patients. We also identify interacting proteins of TMCO1, which include endoplasmic reticulum-resident proteins involved in Ca^2+^ regulation and proteins directly involved in nucleocytoplasmic transport. Interacting proteins included nuclear transport proteins and TMCO1 was shown to have both nuclear and endoplasmic reticulum localisation in MDA-MB-231 basal breast cancer cells. These studies also define a role for TMCO1 in the regulation of breast cancer cells in their sensitivity to BCL-2/MCL-1 inhibitors, analogous to the role of inositol 1,4,5-triphosphate receptors in the regulation of cell death pathways activated by these agents.

## Introduction

The endoplasmic reticulum is a key regulator of cellular protein and lipid homeostasis. Extrinsic factors and events can trigger endoplasmic reticulum stress responses, which may in turn activate a variety of cell death pathways [1]. The endoplasmic reticulum is also vital in intracellular free calcium (Ca^2+^) signalling through its role as the major intracellular Ca^2+^ store in mammalian cells. Endoplasmic reticulum Ca^2+^ stores are dynamic with well-characterised mechanisms to release Ca^2+^ after cell activation such as via inositol 1,4,5-triphosphate receptors (IP_3_Rs). IP_3_Rs release stored intracellular Ca^2+^ from the endoplasmic reticulum following activation of phospholipase C-coupled G-protein coupled receptors and the subsequent generation of inositol 1,4,5-trisphosphate (IP_3_) [2].

In cancer cells, remodelling of endoplasmic reticulum Ca^2+^ homeostasis is particularly significant in the context of sensitivity to death stimuli [3–5]. This remodelling may occur through changes in the expression or activity of IP_3_Rs [5]. Many proteins linked to cell death pathway alterations in cancer cells interact with and modulate IP_3_R activity and subsequently endoplasmic reticulum Ca^2+^ levels. These IP_3_R-interacting proteins and modulators include members of the Bcl-2 family of anti-apoptotic proteins [4, 6]. The binding of BCL-2 and MCL-1 to IP_3_Rs enhances the sensitivity of these channels to IP_3_, increasing the opening probability of these channels and facilitating Ca^2+^ loss or “leak” from the endoplasmic reticulum [7, 8]. Consequences of this include a reduction in endoplasmic reticulum Ca^2+^ levels that result in the generation of sub-maximal Ca^2+^ signals that are insufficient to activate apoptosis typically induced by death-inducing stimuli [9, 10]. This remodelling of endoplasmic reticulum Ca^2+^ levels could be therapeutically exploited, as demonstrated in small cell lung cancer cell lines, where a peptide inhibitor of the BCL-2-IP_3_R interaction enhances the apoptotic effect of navitoclax (ABT-263), a BCL-2/BCL-xL inhibitor [11].

A more recently identified regulator of endoplasmic reticulum Ca^2+^ levels is transmembrane coiled-coil domains 1 (TMCO1). TMCO1 facilitates the loss of Ca^2+^ when endoplasmic reticulum Ca^2+^ store levels become excessive, effectively functioning as a “leak” channel [12]. In the context of cancer, *TMCO1* expression appears to be cancer-specific, with higher expression in gliomas, lung, colon and ovarian cancers and a downregulation in urothelial cancers [13–17]. Although there has been increasing interest in the possible roles of TMCO1 in various cell types, in contrast to IP_3_Rs, little is known about the function, expression pattern and interacting partners of TMCO1, particularly in breast cancer cells. In many ways, TMCO1 mimics some of the features of leaky IP_3_Rs, which are linked to the remodelling of cell death pathways in cancer cells [10, 18]. Due to the recency of TMCO1’s association with endoplasmic reticulum Ca^2+^ regulation and a lack of studies in breast cancer, we sought to evaluate the expression, interacting proteins and consequences of TMCO1 inhibition on Ca^2+^ signalling and cell death in breast cancer cells.

## Results

### TMCO1 expression is increased in breast cancer and associated with poorer survival in node-positive basal breast cancer

**Fig. 1:**
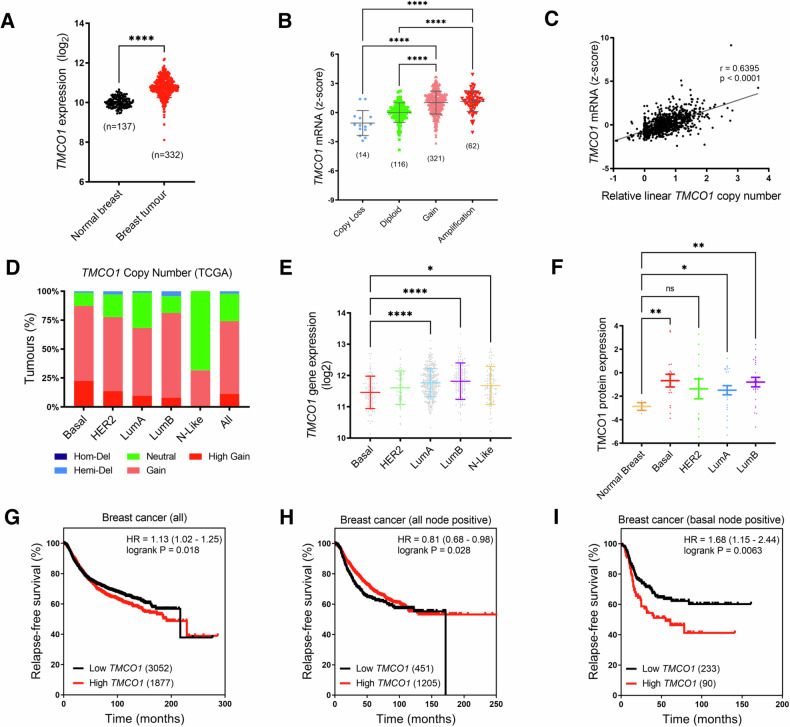
*TMCO1* gene copy number and expression are increased in breast cancer and higher levels are associated with poorer survival in node-positive basal breast cancer. **A** *TMCO1* gene expression in breast tumours compared to normal breast tissues using patient cDNA array data derived from MERAV. ****P < 0.0001, Welch’s t-test **B** *TMCO1* mRNA levels in TCGA breast cancer patients stratified to the gene copy number status. Sample sizes for individual gene copy number groups are shown in the brackets. ****P < 0.0001 (one-way ANOVA, Kruskal-Wallis test) **C** Spearman’s correlation analysis assessing the correlation between *TMCO1* mRNA levels and *TMCO1* copy number in the TCGA dataset. **D** Proportion of *TMCO1* copy number alterations within different breast cancer molecular subtypes in the TCGA dataset. HER2 - HER2-enriched, LumA - Luminal A, LumB - Luminal B, N-like - Normal-like, Hom-Del - Homozygous deletion, Hemi-Del - Hemizygous deletion. **E** *TMCO1* gene expression levels in breast cancer molecular subtypes. **F** TMCO1 protein expression in breast cancer molecular subtypes compared to normal breast tissue samples. ****P < 0.0001, **P < 0.01, *P < 0.05, ns not significant (one-way ANOVA, Tukey’s test) Error bars represent mean ± S.D. **G**–**I** Kaplan-Meier plots showing the association between low or high TMCO1 expression (patient numbers within brackets) with relapse-free survival in all breast cancers, all node-positive breast cancers and node-positive basal breast cancers respectively. *P*-values and hazard ratios (HR) are shown in the graphs.

We accessed several publicly available breast cancer patient databases using a web-based gene expression analysis tool (MERAV) [19] and showed that *TMCO1* mRNA expression is higher in breast tumours compared to normal breast tissues (Fig. 1A). Approximately 70% of TCGA breast cancer patients had a gain in at least one copy of the *TMCO1* gene (Fig. 1B). *TMCO1* expression was higher in the *TMCO1* copy number gain and amplified groups compared to diploid and copy loss groups. This relationship between *TMCO1* gene expression and gene copy number was also apparent in the *METABRIC* patient dataset (Supplementary Fig. 1A). *TMCO1* mRNA expression was positively correlated with gene copy number (Spearman’s correlation R-value = 0.6395) (Fig. 1C), suggesting that the increased TMCO1 expression in breast cancers is the result of gene copy number alterations.

Breast cancer subtypes differ not only in their gene and molecular expression profile but are also predictive of patient survival [20, 21]. We assessed if *TMCO1* gene copy number and expression was associated with any specific breast cancer molecular subtype. *TMCO1* copy number gains were seen across all molecular subtypes in both TCGA (Fig. 1D) and METABRIC patient datasets (Supplementary Fig. 1B) with no clear association with subtype. Basal breast cancers had a modest but significantly lower level of *TMCO1* mRNA than Luminal A and Luminal B in both data sets (Fig. 1E and Supplementary Fig. 1C), but there was no strong association between *TMCO1* mRNA levels and breast cancer molecular subtype. Indeed, TMCO1 protein was significantly elevated in the luminal A, luminal B and basal breast cancer subtypes compared to normal breast (Fig. 1F), indicating that elevated TMCO1 may be a general feature of breast cancers. Finally, we assessed whether *TMCO1* expression was correlated with breast cancer patient relapse-free survival using a log-rank test. *TMCO1* expression had a modest association with relapse-free survival of all breast cancer patients overall (Fig. 1G) and in node-positive breast cancer patients (Fig. 1H). To better identify subsets of node-positive breast cancer patients for which TMCO1 expression had potential prognostic value, node-positive breast cancer patients were further stratified into PAM50 molecular subtypes. There was no correlation between *TMCO1* expression and relapse-free survival of breast cancer patients of the Luminal A and B and HER2 subtypes (Table S1). However, higher *TMCO1* expression was significantly correlated with poorer survival in node-positive basal breast cancer patients (Fig. 1I). These results suggest that while *TMCO1* expression is not associated with any specific breast cancer subtype, TMCO1 may play a more significant role in the basal subtype given its association with poorer survival. As such, we explored the functional role of TMCO1 using basal breast cancer cell lines in the studies below.

### Effect of TMCO1 silencing on calcium signalling in basal breast cancer cells

**Fig. 2:**
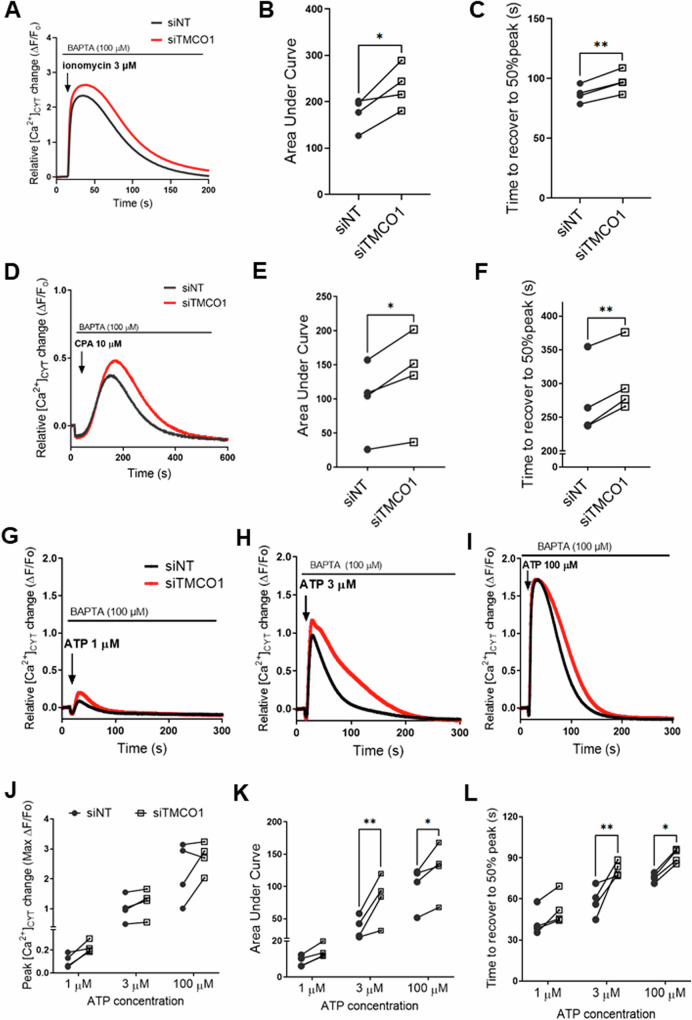
Effect of *TMCO1* silencing on calcium signalling in MDA-MB-231 breast cancer cells. **A** Trace shows the mean relative cytosolic calcium ([Ca^2+^]_CYT_) increase as a result of ionomycin addition (3 µM) in GCaMP6m-MDA-MB-231 cells transfected with NT (siNT) or TMCO1 siRNA (siTMCO1). **B**, **C** Area under curve of [Ca^2+^]_CYT_ increase following ionomycin addition from 15 to 200 s and recovery time following the maximum [Ca^2+^]_CYT_ increase respectively. **D** Mean relative [Ca^2+^]_CYT_ increase with CPA (10 µM) addition. **E**, **F** Area under curve of [Ca^2+^]_CYT_ increase following CPA addition from 15 to 600 s and recovery time following maximum [Ca^2+^]_CYT_ increase respectively. **G**–**I** Traces show mean relative [Ca^2+^]_CYT_ increase following ATP 1 µM, 3 µM and 100 µM addition respectively. **J**–**L** Peak [Ca^2+^]_CYT_ increase, area under curve and recovery time following the maximum [Ca^2+^]_CYT_ increase as a result of ATP addition respectively. All data points shown represent mean of triplicate wells (*n* = 4). Statistical analyses for (**B**, **C**, **E**, **F**) were done using paired t-test and **J**,**K**,**L** were analysed using a two-way ANOVA (Bonferroni’s test). **P < 0.01, *P < 0.05.

TMCO1 functions as a Ca^2+^ load-activated Ca^2+^ leak channel in HeLa cells and osteoblasts [12, 22]. To define the role of TMCO1 in Ca^2+^ homeostasis in the highly metastatic MDA-MB-231 basal breast cancer cells, we assessed the consequences of *TMCO1* silencing (Supplementary Fig. 2) on Ca^2+^ signals associated with endoplasmic reticulum Ca^2+^ stores. *TMCO1* silencing increased cytosolic Ca^2+^ increases induced by the Ca^2+^ ionophore and endoplasmic reticulum Ca^2+^ mobilising agent ionomycin [23] (Fig. 2A), i.e. increased area under curve (Fig. 2B) and prolonged recovery to baseline Ca^2+^ levels (Fig. 2C). To more specifically assess the effect of *TMCO1* silencing on endoplasmic reticulum Ca^2+^ levels, MDA-MB-231 cells were treated with cyclopiazonic acid (CPA), a pharmacological inhibitor of the endoplasmic reticulum Ca^2+^ reuptake pump - sarco/endoplasmic reticulum Ca^2+^-ATPase (SERCA) [24]. *TMCO1* silencing also increased the endoplasmic reticulum Ca^2+^ levels with CPA treatment (Fig. 2D–F). We also assessed the effect of *TMCO1* silencing on G protein-coupled endoplasmic reticulum Ca^2+^ release due to purinergic receptor activation via ATP addition in the absence of extracellular Ca^2+^ (extracellular BAPTA). *TMCO1* silencing did not significantly increase peak Ca^2+^ levels after ATP-mediated Ca^2+^ store release (Fig. 2G–J) but did increase both the area under curve and recovery rate (Fig. 2K, L). These results are consistent with the literature, confirming the role of TMCO1 in regulating endoplasmic reticulum Ca^2+^ signalling in breast cancer cells.

### IP LC-MS/MS analysis identifies enrichment in nuclear transport proteins interacting with TMCO1

**Fig. 3:**
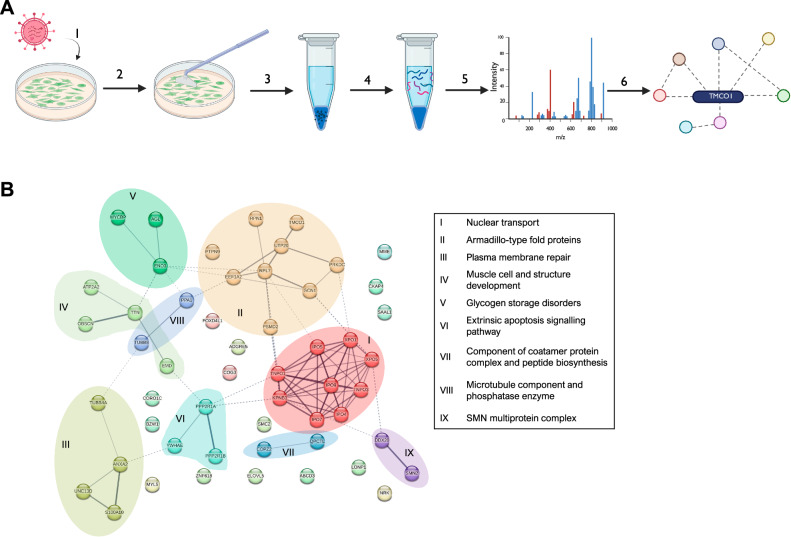
IP-MS/MS analysis of TMCO1-interacting proteins. **A** Identification of proteins interacting with FLAG-TMCO1 expressed in MDA-MB-231 cells. Lentiviral transduction was used to generate MDA-MB-231 cells overexpressing FLAG-tagged TMCO1 (1) Cells were lysed (2) and FLAG-TMCO1 and interacting proteins were co-immunoprecipitated using anti-FLAG beads (3), trypsin digestion was performed (4) and resulting peptides were analysed by liquid chromatography-tandem mass spectrometry (5). Acquired mass spectrometry spectra was searched against human databases and TMCO1 interactome was identified (6). Figure was created using BioRender.com. **B** Gene clustering analysis of proteins interacting with TMCO1 using StringDB (confidence level: medium (score 0.4), FDR = 5%). Nine clusters (I – IX) are identified and visualised as interacting proteins with the same colour and same background shade. Nodes represent individual proteins identified in the LC-MS/MS. Line thickness between proteins indicate the strength of the evidence for the interaction from available data.

To gain insights into the function of TMCO1 in basal breast cancer cells we performed proteomic analyses. MDA-MB-231 cells stably-overexpressing FLAG-tagged TMCO1 were generated, immunoprecipitated with FLAG beads followed by mass spectrometry (LC-MS/MS) (see Fig. 3A; method previously described in [25]). Following removal of background, a total of 53 proteins were obtained (see Table S2). As expected, several endoplasmic reticulum-resident proteins involved in Ca^2+^ regulation, protein processing and translocation were identified (ATP2A2, COG3, COPZ2, CORO1C, ELOVL5, GCN1, LONP1, PSMD2, PTPN9). Proteins directly involved in nucleocytoplasmic transport were also identified as TMCO1 interactors (IPO4, IPO5, IPO7, IPO9, KPNB1, TNPO1, TNPO3, XPO1, XPO5). In particular, highest peptide numbers were identified for IPO7 and KPNB1. To further explore biological processes associated with proteins interacting with TMCO1, we performed gene ontology (GO) functional enrichment analysis for the list of identified proteins using StringDB (Fig. 3B). The GO analysis revealed a broad functional enrichment of the TMCO1 interactome in nucleocytoplasmic carrier activity (FDR 0.0182). To further delineate the roles of all the interacting proteins, we performed Markov clustering (inflation parameter 1.8) [26] to the list and obtained 9 clusters (Fig. 3B), where 6 out of the 9 clusters are associated with GO functional enrichment with 3 genes and above. Cluster I comprised the 9 genes previously mentioned, which are associated with nuclear transport. Cluster II comprised genes of the Armadillo-type fold structural domain, with broad roles in intracellular transport and localisation [27]. Meanwhile, Cluster III was enriched for genes involved in regulation of plasma membrane repair (GO: 1905686), cluster IV was involved in muscle cell and structure development (GO: 0055001), cluster V comprised genes associated with glycogen storage disorders and cluster VI was associated with regulation of the extrinsic apoptotic signalling pathway (GO:2001241). No significant functional enrichments were detected for clusters VII (comprising COPZ2 and QPCTL) and VIII (comprising PPA1 and TUBB8). Finally, cluster IX was enriched in the SMN (survival motor neuron) complex cellular component (GO:0032797), a multiprotein complex essential in the processing and transport of ribonucleoproteins. These observations suggested that TMCO1 could be involved in nuclear transport in breast cancer cells and further exploration was warranted.

#### Assessing the role of TMCO1 in nuclear transport

**Fig. 4:**
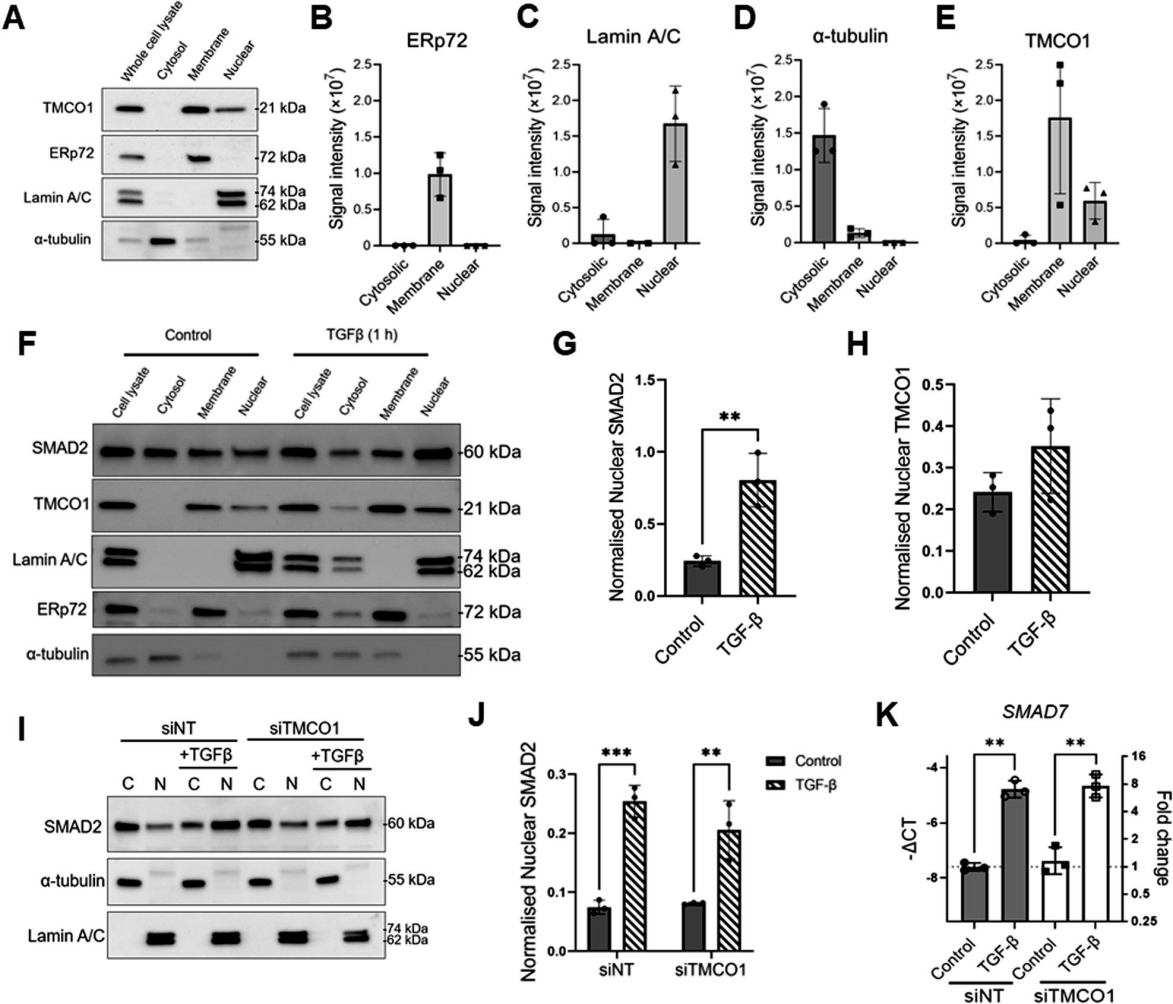
Assessment of TMCO1 subcellular localisation and role in TGFβ-mediated nuclear transport. **A** Cell fractionation of MDA-MB-231 breast cancer cells into cytosolic, membrane and nuclear fractions. Markers for fractions are: ERp72 – membrane, Lamin A/C – nuclear, α-tubulin – cytosolic. Whole cell lysate was included for normalisation. **B**–**E** Densitometric analysis of cell fractionation blots for ERp72, Lamin A/C, α-tubulin and TMCO1 respectively. (*n* = 3) **F** Representative blot shows the effect of 1 h TGFβ treatment (10 ng/mL) on SMAD2 and TMCO1 nuclear translocation compared to control. Loading controls used were Lamin A/C (nuclear), ERp72 (membrane) and α-tubulin (cytosolic). **G**, **H** Densitometric analyses showing the effect of TGFβ on SMAD2 and TMCO1 nuclear translocation. Nuclear SMAD2 and TMCO1 blots were normalised to corresponding Lamin A/C blots (*n* = 3) **P < 0.01 Unpaired t-test. **I** Representative blot showing the effect of TMCO1 silencing (siTMCO1) on TGFβ-mediated SMAD2 translocation from the cytosol (**C**) to the nucleus (N) compared to non-targeting control (siNT). **J** Densitometric analysis showing the effect of siTMCO1 on nuclear SMAD2. Each bar represents the fraction of nuclear SMAD2 of that treatment group relative to the total SMAD2, normalised to the fraction of Lamin A/C of the same treatment group. (*n* = 3) **K** Effect of TMCO1 silencing on TGFβ-induced SMAD7 mRNA levels. Bar graph shows -ΔC_T_ (left axis) and fold change in SMAD7 mRNA (right axis) compared to control (siNT group). (*n* = 3) ***P < 0.001, **P < 0.01 two-way ANOVA with Tukey’s post-hoc test (**J** and **K**). Error bars represent mean ± S.D.

Although TMCO1 is mostly localised to the endoplasmic reticulum [12], immunolabelling studies in the human ocular optic nerve and the SH-SY5Y cell line reported that TMCO1 is co-localised with nucleoli within the nucleus in these cell types [28]. This suggests that the subcellular distribution of TMCO1 may be cell-type specific and/or dynamic. Given our LC-MS/MS studies showed an enrichment in the TMCO1 interactome in nuclear transport, we evaluated the subcellular localisation of TMCO1 in parental MDA-MB-231 breast cancer cells by performing cell fractionation studies. As shown in Fig. 4A–D, minimal contamination between the different fractions was observed in our fractionation experiments. TMCO1 was mostly found in the membrane fraction with some TMCO1 also detected in the nuclear fraction (Fig. 4A, E).

Our LC-MS/MS studies showed that TMCO1 interacts with several nuclear import and export proteins, with the top interacting protein being KPNB1 (karyopherin subunit beta 1) and IPO7 (importin 7). *IPO7* expression is elevated in breast cancer and is associated with poorer patient survival and *IPO7* expression inhibition promotes the drug sensitivity of triple-negative breast cancer cells [29]. IPO7 is involved in the non-canonical nuclear import of cargo proteins including SMAD transcription factors [30]. SMAD2, SMAD3 and SMAD4, translocate to the nucleus when the TGFβ signalling pathway is activated [31]. Given the link between the TGFβ pathway and breast cancer invasion and metastasis [32], we explored the possible role of TMCO1 in TGFβ-mediated nuclear transport. We first assessed whether TMCO1 itself could be induced to translocate to the nucleus in response to TGFβ. TGFβ treatment significantly increased the translocation of SMAD2 from the cytosol into the nucleus (Fig. 4F, G). However, there was no significant increase in nuclear TMCO1 following TGFβ treatment (Fig. 4F, H), suggesting that TMCO1 does not translocate to the nucleus under these conditions. We next assessed the effect of *TMCO1* silencing on TGFβ-induced nuclear translocation of SMAD2. However, we did not observe a significant change in SMAD2 nuclear translocation due to T*MCO1* silencing compared to control (Fig. 4I, J). To further assess whether TMCO1 had any effect on TGFβ-mediated signalling, we assessed the mRNA expression of *SMAD7*, a negative feedback regulator which is upregulated in response to TGFβ [33]. *SMAD7* mRNA levels are increased with TGFβ treatment, however, there was no significant difference in *SMAD7* levels between *TMCO1* silenced cells and NT control (Fig. 4K). Collectively, our data showed that TMCO1 is not involved in TGFβ-mediated nucleocytoplasmic transport of SMAD2.

### TMCO1 silencing promotes cell death in basal breast cancer cells treated with selective apoptosis inducers

**Fig. 5:**
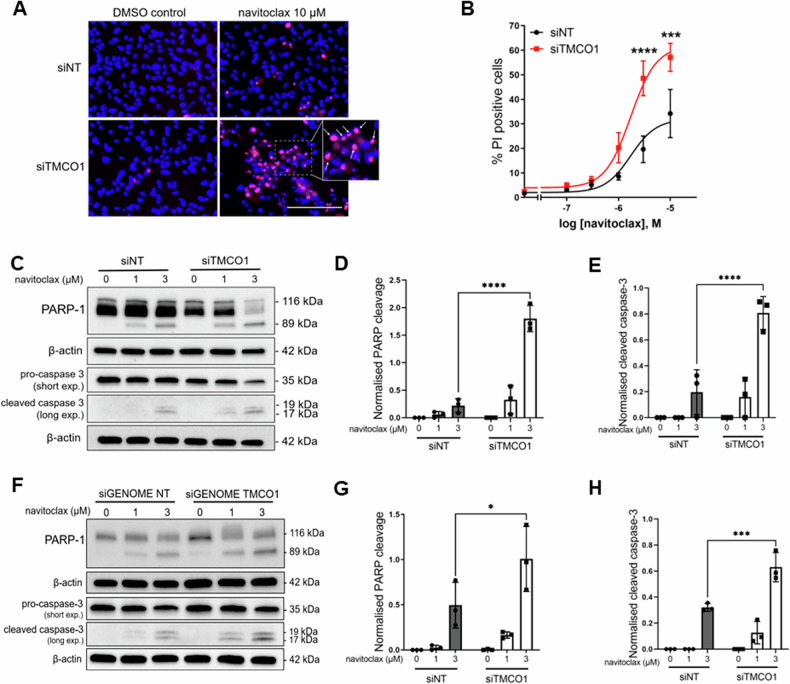
*TMCO1* silencing augments apoptosis induced by navitoclax in MDA-MB-231 cells. **A** Representative fluorescence microscopy images showing increased cell death in TMCO1-silenced GCaMP6m-MDA-MB-231 cells after 24 h navitoclax (10 µM) treatment compared to 0.1% DMSO control. Cell nuclei were stained with Hoescht33342 (blue). Positive scoring for propidium iodide (PI; magenta) staining represents cell death (annotated by white arrows in image inset). Scale bar = 150 µM **B** Concentration-effect curve showing %PI positivity at different concentrations of navitoclax. Data points represent mean ± S.E.M (*n* = 3). **C** Representative immunoblots showing increased PARP-1 and caspase-3 cleavage as a result of *TMCO1* silencing in cells treated with navitoclax. **D**, **E** Densitometric analyses showing the effect of *TMCO1* silencing on PARP-1 and caspase-3 cleavage with navitoclax treatment. **F** Representative immunoblots showing the effects of siGENOME *TMCO1* silencing on PARP-1 and caspase-3 cleavage in GCaMP6m-MDA-MB-231 cells. **G**, **H** Densitometric analyses of the siGENOME immunoblots on PARP-1 and caspase-3 cleavage respectively. Bar graphs show mean ± S.D. (*n* = 3) *P < 0.05, ***P < 0.001, ****P < 0.0001 (two-way ANOVA with Sidak’s post-hoc test).

The LC-MS/MS studies identified a cluster of proteins associated with regulation of the extrinsic apoptotic signalling pathway (PPP2R1A, PPP2R1B, YWHAE). This along with extensive work linking regulators of endoplasmic reticulum Ca^2+^ homeostasis and cell death [8, 34–36], highlighted the importance of considering TMCO1 in the regulation of cell death pathways in basal breast cancer cells. Regulators of endoplasmic reticulum Ca^2+^ leak such as Bax-inhibitor 1 (BI-1) have a cytoprotective role, whereby their inhibition results in an increased sensitivity to cell death inducers [37, 38]. An analogous role for TMCO1 has not yet been explored. We therefore assessed the potential of *TMCO1* silencing to promote cancer cell death induced by agents that are sensitive to endoplasmic reticulum Ca^2+^ levels. MDA-MB-231 cells were treated with the BCL-2 inhibitor, navitoclax at increasing concentrations. As shown in Fig. 5A, B, navitoclax significantly induced cell death in MDA-MB-231 cells transfected with NT control at concentrations of 3 µM ( ~ 20%) and 10 µM ( ~ 35%). With *TMCO1* silencing, the percentage of cell death significantly increased at navitoclax concentrations of 3 and 10 µM. As navitoclax is a known apoptosis inducer, we verified whether the death-enhancing effect of *TMCO1* silencing was via apoptosis through immunoblot-based assessment of PARP-1 and caspase-3 cleavage. Navitoclax treatment induced PARP-1 and caspase-3 cleavage, and *TMCO1* silencing enhanced this effect (Fig. 5C–E). To confirm that these effects were not due to a clonal effect in the GCaMP6m-MDA-MB-231 cell lines used in these studies, we repeated these studies in parental MDA-MB-231 cells. *TMCO1* silencing in the parental cells also significantly increased the percentage of cell death with navitoclax (Supplementary Fig. 3A), and increased PARP-1 and caspase-3 cleavage (Supplementary Fig. 3B–D). As shown in Fig. 5F–H, we also observed a promotion of PARP-1 and caspase-3 cleavage with *TMCO1* siGENOME siRNAs.

**Fig. 6:**
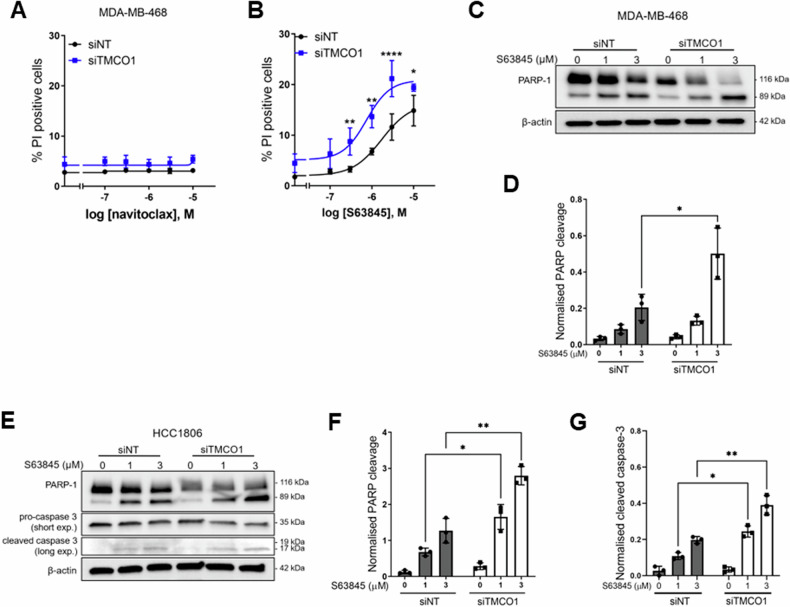
*TMCO1* silencing promotes cell death to MCL-1 inhibition in MDA-MB-468 and HCC1806 basal breast cancer cells. **A**, **B** Concentration-effect curve showing the effect of *TMCO1* silencing on %PI positive cells in MDA-MB-468 cells treated with navitoclax and S63845 (MCL-1 inhibitor) respectively. **C**, **D** Representative immunoblot and densitometric analysis showing increased PARP-1 cleavage with *TMCO1* silencing in MDA-MB-468 cells treated with S63845. **E** Representative immunoblots showing the effect of *TMCO1* silencing on PARP-1 and caspase-3 cleavage in HCC1806 cells treated with S63845. **F**, **G** Densitometric analyses of immunoblots on PARP-1 and caspase-3 respectively in HCC1806 cells. All bar graphs show mean ± S.D. (*n* = 3) *P < 0.05, **P < 0.01, ****P < 0.0001 (two-way ANOVA with Sidak’s post-hoc test).

To further explore the ability of *TMCO1* silencing to promote death in basal breast cancer cells, we performed cell death studies using other basal breast cancer cell lines that have been previously assessed in the context of Bcl-2 inhibition [39–41]. MDA-MB-468 cells are less sensitive to navitoclax [42] and our studies indeed showed no cell death at any concentration of navitoclax assessed (Fig. 6A). *TMCO1* silencing (Supplementary Fig. 4A) also did not restore sensitivity to navitoclax in these cells. MDA-MB-468 cells are, however, sensitive to MCL-1 inhibition [39, 41]. Like BCL-2, MCL-1 also facilitates pro-survival endoplasmic reticulum Ca^2+^ signals through interaction with IP_3_R [8]. Thus, we evaluated the ability of *TMCO1* silencing to promote cell death mediated by the MCL-1 inhibitor, S63845 [43]. S63845 treatment had a concentration-dependent effect on cell death in MDA-MB-468 cells (Fig. 6B). *TMCO1* silencing significantly increased cell death induced by S63845. TMCO1 silencing also promoted PARP cleavage induced by S63845 (Fig. 6C, D). To validate this phenomenon, we assessed the ability of *TMCO1* silencing to promote apoptosis to MCL-1 inhibition in another invasive basal breast cancer cell line, HCC1806 (Supplementary Fig. 4B). S63845 treatment induced apoptosis in HCC1806 cells, as shown by increased PARP-1 and caspase-3 cleavage, and these effects were significantly increased with *TMCO1* silencing (Fig. 6E–G). Collectively, these results show that the endoplasmic reticulum Ca^2+^ leak channel TMCO1 is a regulator of apoptosis in basal breast cancer cell lines induced by agents previously linked to endoplasmic reticulum Ca^2+^ levels.

## Discussion

Regulators of endoplasmic reticulum Ca^2+^ homeostasis such as IP_3_Rs represent important potential targets to induce cancer cell death. In this study, we explored the role of the endoplasmic reticulum Ca^2+^ leak channel TMCO1 in breast cancer. Consistent with previous reports, we found higher *TMCO1* expression in breast cancer compared to normal breast tissues [15, 16]. Given the prognostic relevance of breast cancer molecular subtypes, we further explored the association between *TMCO1* expression with subtypes and found elevated *TMCO1* expression in all subtypes. We also showed a positive correlation between *TMCO1* gene copy number and mRNA expression levels. Finally, we observed that higher *TMCO1* expression is associated with poorer survival in node-positive basal breast cancer patients but not other subtypes, implicating a potential importance of TMCO1 in more advanced basal breast cancers, for which there is a pressing clinical need for more effective therapies [44]. Future studies assessing the transcriptome profile of basal breast cancer patients with high versus low *TMCO1* expression could provide a gene signature associated with *TMCO1* overexpression, and new insights into the possible contribution of TMCO1 to basal breast cancer metastasis.

TMCO1 functions as a Ca^2+^-load activated channel, which forms active homotetramers and opens in response to overfilling of endoplasmic reticulum Ca^2+^ load. However, under basal conditions, TMCO1 also functions as a leak channel, even in the absence of an overfilled Ca^2+^ store [12]. Indeed, our study showed that under basal conditions, *TMCO1* silencing increased endoplasmic reticulum Ca^2+^ stores reflected by the increased amount of Ca^2+^ released with ionomycin, CPA and ATP treatment in the absence of extracellular Ca^2+^. These findings are consistent with observations in other cell types [12, 14, 16, 22, 45]. We also found delayed recovery of cytosolic Ca^2+^ levels back to baseline following IP_3_R activation in *TMCO1* silenced cells, which could also be a consequence of increased endoplasmic reticulum Ca^2+^ store levels, but would be further enhanced by decreased Ca^2+^ re-sequestration by endoplasmic reticulum SERCA pumps. Recovery of Ca^2+^ signals in prostate cancer cells is dependent on SERCA [46] and the activity of SERCA could be altered with *TMCO1* silencing as our LC-MS/MS studies identified SERCA2 (*ATP2A2*) as an interacting partner with TMCO1. This potential association between TMCO1 and SERCA2 activity should be explored in future studies.

Using FLAG-tagged TMCO1-overexpressing MDA-MB-231 cells to identify potential interacting partners using LC-MS/MS revealed a strong association with nucleocytoplasmic transport proteins. An earlier study, screening for cargo proteins of the nuclear transport protein importin-α in murine embryonic fibroblasts, also identified TMCO1 as an interacting protein [47]. Two of the top interactors identified in all three pulldowns, ANXA2 and IPO7 are also implicated in invasive breast cancers [29, 48, 49]. Our cell fractionation studies in parental MDA-MB-231 cells showed that in addition to the membranous compartment, TMCO1 is also localised to the nucleus under basal conditions, consistent with another study showing that TMCO1 is co-localised within the nucleoli in SH-SY5Y and human ocular nerve cells [28]. These results further support our proteomics data that TMCO1 may have nuclear-related functions. Given these findings, we then focused our investigation on IPO7-related pathways, specifically SMAD2 nuclear translocation in response to TGFβ. However, our investigations showed that the subcellular distribution of TMCO1 was not affected during TGFβ stimulation, nor did *TMCO1* silencing affect TGFβ-mediated SMAD2 signalling. While these data collectively suggest that TMCO1 is not involved in TGFβ-mediated SMAD2 translocation, the possibility that TMCO1 is involved in other nuclear transport pathways cannot be excluded. Indeed, in addition to SMAD transcription factors, IPO7 is also a nuclear transporter for various other proteins including extracellular-signal regulated kinase (ERK) and Yes-associated protein (YAP) [50–52]. Future studies should focus on characterising the role of TMCO1 in the nuclear transport of these proteins. TMCO1 knockdown in mouse glial cells increases ERK signalling [53]. However, as our LC-MS/MS studies showed that TMCO1 interacts with various other nuclear transport proteins, it is likely that TMCO1 itself is a cargo protein. Indeed, other proteins that are transported via different importins have been identified [54–56]. In addition, some nuclear transport receptors also function as chaperones for proteins implicated in cancers such as PTEN and eIF5A [57, 58]. Given the complexity and highly redundant functions of the nuclear transport proteins identified as TMCO1 interactors, more studies are clearly required to delineate the functional association between TMCO1 and the nuclear transport receptors identified in our study.

Our GO clustering analysis of the TMCO1 interactome also revealed an association with other biological pathways largely related to maintenance of cellular homeostasis and intracellular protein/ribosomal transport, which is not surprising given TMCO1’s role in maintaining Ca^2+^ homeostasis. We also identified a cluster associated with the extrinsic apoptotic pathway involving PPP2R1A, PPP2R1B and YWHAE, proteins dysregulated in breast cancer [59, 60]. Consistent with a role in apoptosis, previous studies showed that *TMCO1* knockout or silencing directly induced apoptosis in ovarian granulosa and cervical, colorectal and glioma cancer cell lines [15, 16, 45, 61]. However, we found that *TMCO1* silencing on its own did not induce apoptosis in MDA-MB-231 and MDA-MB-468 cells. Instead, *TMCO1* silencing enhanced apoptosis to navitoclax in MDA-MB-231 cells, and to S63845 in MDA-MB-468 and HCC1806 basal breast cancer cells. The variable sensitivities of these breast cancer cell lines to navitoclax or S63845 are consistent with other studies showing a lack of correlation between breast cancer subtype and BCL-2/MCL-1 dependencies; instead sensitivity to these drugs are better correlated with the expression profile of BCL-2 family proteins specific to each cell line [39, 42]. Our finding that *TMCO1* silencing only promoted apoptosis to agents to which the breast cancer cells are already sensitive to suggests a likely role for TMCO1 in regulating stress tolerance responses in breast cancer cells. Consistent with this explanation, previous studies showed that *TMCO1* knockdown increases levels of the endoplasmic reticulum stress sensor, inositol-requiring enzyme 1 (IRE1α) in other cell types [45, 61, 62]. Interestingly, members of the BCL-2 family can also associate with IRE1α, constituting a model of “stress rheostat” in which apoptosis may be triggered during irreversible cellular stress conditions [63, 64]. Furthermore, in glioma cell lines and ovarian granulosa cells, TMCO1 knockdown is associated with decreased levels of BCL-2 [15, 45], potentially affecting the BH3 profile and therefore sensitivity to BCL-2 inhibitors used in our study. In summary, we identified that TMCO1 gene and protein expression is increased in all breast cancer subtypes and higher *TMCO1* expression is associated with poorer survival in more advanced basal breast cancers. We also characterised the role of TMCO1 in the regulation of endoplasmic reticulum Ca^2+^ signals in a basal breast cancer cell line. Using LC-MS/MS proteomics, we found a novel interaction between TMCO1 and nuclear transport proteins, including KPNB1 and IPO7, which are associated with several cancers. Finally, we also show that *TMCO1* silencing can promote apoptosis to navitoclax and S63845, inhibitors of the BCL-2 anti-apoptotic protein family in basal breast cancer cell lines. Given the increasing interest in the clinical use of BCL-2/MCL-1 inhibitors, the findings from our study now provide another avenue towards exploring potential combination treatment involving BCL-2/MCL-1 inhibitors and TMCO1 inhibition for treating basal breast cancers.

## Materials and methods

### Cell culture

Parental MDA-MB-231 cells were sourced from ATCC and MDA-MB-468 cells were obtained from the Brisbane Breast Bank, UQCCR, Brisbane, Australia. Both cell lines were cultured in DMEM containing 10% FBS and 4 mM L-glutamine. GCaMP6m-MDA-MB-231 were developed as previously described [65] and cultured in DMEM containing 10% FBS, 4 mM L-glutamine and 400 µg/mL hygromycin B. HCC1806 cells were a kind gift from Kum-Kum Khanna (QIMR Berghofer, Brisbane, Australia) and were cultured in RPMI-1640 containing 10% FBS. Cell line authenticity was verified by short tandem repeats (STR) DNA profiling at QIMR Berghofer using the GenePrint 10 System (Promega, Madison, WA, USA). Cells were maintained at 37 °C in a 5% CO_2_ humidified incubator and tested for mycoplasma contamination bi-annually using the MycoAlert Mycoplasma Detection kit (Lonza, Basel, Switzerland).

### Generation of FLAG-tagged TMCO1 overexpressing MDA-MB-231 cells

Human *TMCO1* variant D (# 46827061, Applied Biological Materials, Richmond, Canada) was amplified and cloned with a N-terminal FLAG sequence into pcDH-EF1-FHC lentiviral vector (Addgene #64874). Lentiviral particles were generated in HEK293T cells transfected with second generation plasmids (Addgene # 8455 and #8454) using Lipofectamine 2000. MDA-MB-231 cells were transduced in the presence of 8 µg/mL of polybrene. The viral media was replaced after 24 h and cells were selected with 2 µg/mL of puromycin 48 h post transduction.

### TMCO1 gene copy number and expression analysis

*TMCO1* gene expression in primary breast tumours compared to normal breast tissues was assessed using normalised cDNA array data on the Metabolic gEne RApid Visualizer web-based tool [19]. Analyses done using this online tool were based on a collection of normal tissue and primary breast tumour samples from the Expression Project for Oncology (GSE2109), Human Body Index (GSE7307) and the Gene Expression Omnibus database as described in their original paper [19]. TMCO1 protein expression in the TCGA breast cancer database was stratified to the Prosigna Prediction Analysis of Microarray 50 (PAM50) gene signature profiling of breast cancer molecular subtypes using mass-spectrometry data generated by Mertins et al. [66]. *TMCO1* gene copy number data from the TCGA and METABRIC patient cohorts were downloaded from cBioportal (https://www.cbioportal.org/) and graphed using GraphPad Prism 9.0.

### Kaplan-Meier survival analysis

Patient survival analyses were done using Kaplan-Meier Plotter (https://kmplot.com/analysis/). Relapse-free survival curves of breast cancer patients were stratified to high or low expression of *TMCO1* (Affymetrix ID: 208715_at) using the “auto-select best cut-off” function. Pooled patient survival data from the following datasets were used in the analysis: E-MTAB-365, GSE11121, GSE12276, GSE1456, GSE16391, GSE16446, GSE16716, GSE17705, GSE19615, GSE2034, GSE20685 and GSE20711. Patient sample sizes, log-rank *P-*values and hazard ratios (HR) are shown in the figures or figure legends.

### Immunoblotting

Protein lysates were prepared by incubating cells with cold protein lysis buffer containing protease inhibitor mixture as described previously [65]. Gel electrophoresis was performed using Mini-PROTEAN TGX 4-15% Pre-Cast Gels (#4561084, Bio-Rad, Hercules, CA, USA) and protein samples were transferred onto a PVDF membrane. Membranes were blocked with 5% skim milk in PBS containing 0.1% Tween-20 for 1 h. Proteins of interest were detected using the following antibodies and dilutions: TMCO1 (1:1000, PA5-43350, Invitrogen, Waltham, MA, USA), ERp72 (1:1000, #5033, Cell Signaling Technology (CST), Danvers, MA, USA), Lamin AC (1:4000, #4777, CST), α-tubulin (1:1000, SC-5286, Santa Cruz Biotechnology, Dallas, TX, USA), SMAD2 (1:1000, #5339, CST), PARP (1:1000, #9542, CST), caspase-3 (1:1000, #9662, CST), β-actin (1:10 000, A5441, Sigma-Aldrich, St. Louis, MO, USA). All primary antibodies were incubated overnight at 4 °C, except for β-actin and Lamin A/C, which were incubated for 1 h at room temperature. Membranes were washed using phosphate buffered saline containing 0.1% Tween-20 before a 1 h incubation with either goat-anti-mouse (#170-6516, Bio-Rad) or goat-anti-rabbit (#170-6515, Bio-Rad) HRP-conjugated secondary antibodies (1:10 000 dilution). Protein bands were imaged using a chemiluminescence reagent (SuperSignal^TM^ West Dura Extended Duration Substrate, #34076, Thermo Fisher Scientific, Waltham, MA, USA) and a ChemiDoc^TM^ Touch Imaging System (Bio-Rad). Protein band quantification was done using the ImageLab software (ver. 6.1, Bio-Rad).

### siRNA transfection

Dharmacon ON-TARGETplus SMARTpool siRNAs that comprise a mixture of four rationally designed siRNAs against a single target gene were used in this study. To validate the phenotypic effects of ON-TARGETplus siRNAs, a chemically distinct set of SMARTpool siRNAs (siGENOME) were also used. Cells were seeded into 96-well plates and transfected with siRNA after 24 h. siRNAs used and transfection conditions were: SMARTpool ON-TARGETplus Non-targeting ((NT) (D-001810-10-05, Dharmacon, Horizon Discovery), SMARTpool ON-TARGETplus TMCO1 siRNA (L-013757-02, Dharmacon Inc., Horizon Discovery Biosciences Ltd, Cambridge, UK) at a final concentration of 100 nM using DharmaFECT4 (0.1 µL per well). HCC1806 cells were transfected with 50 nM of ON-TARGETplus siRNA using 0.05 μL of DharmaFECT4 per well. SMARTpool siGENOME *TMCO1* siRNA (M-013757-01, Dharmacon) or SMARTpool siGENOME NT Control siRNA (D-001206-13-05, Dharmacon) were transfected at a concentration of 25 nM using 0.05 μL of DharmaFECT4 per well. RNA was isolated at 48 h and protein was isolated 72–96 h post-transfection to verify *TMCO1* knockdown.

### Imaging and analysis of intracellular calcium changes

Changes in cytosolic Ca^2+^ levels were imaged using the Fluorescence Imaging Plate Reader (FLIPR^TETRA^, Molecular Devices, San Jose, California, USA). GCaMP6m-MDA-MB-231 cells were plated at a density of 4000 cells per well in 96-well black-walled CellBIND microplates (CLS3340, Corning Inc., NY, USA). Post-siRNA transfection (96 h), cells were washed and incubated in physiological salt solution containing nominal Ca^2+^ [67] for 15 min to allow equilibration to room temperature. Plates were loaded into the FLIPR system and reagents containing BAPTA (100 µM) and Ca^2+^-mobilising agents ATP (1, 3 and 100 µM; A6419, Sigma-Aldrich), ionomycin (1 and 3 µM; ALX-450-006-M005, Enzo Life Sciences, Farmingdale, NY, USA) and cyclopiazonic acid (CPA; 10 µM, C1530, Sigma-Aldrich) were added to the cells. Fluorescence intensity (F) changes represent changes in cytosolic Ca^2+^ levels and were measured at 470–495 nm excitation and 515–575 nm emission wavelengths.

For analysis of Ca^2+^ traces, data were exported from ScreenWorks software (Molecular Devices) into Microsoft Excel. Changes in [Ca^2+^]_CYT_ are shown as normalised values (ΔF/F_0_) and calculated using the formula (F – F_0_) ÷ F_0_, where F_0_ represents baseline fluorescence. For assessment of area under curve, individual Ca^2+^ traces for each biological replicate were plotted in GraphPad Prism, and analysis was performed using the “area under curve” function.

### Assessment of cell death using propidium iodide staining and immunoblotting

MDA-MB-231 cells were seeded at 4000 cells per well in FluoroBrite^TM^ DMEM media (A1896701, Thermo Fisher Scientific) into black-walled 96 well microplates (BD Falcon, Corning) and treated with navitoclax (S1001, SelleckChem, Houston, TX, USA) 72 h after siRNA transfection. After 24 h, cells were incubated with a stain solution containing propidium iodide (1 µg/mL) and Hoecsht 33342 (10 µg/mL) for 15 min at 37 °C before imaging with the ImageXpress Micro (Molecular Devices) using the DAPI (excitation: 377/50 nm; emission: 477/60 nm) and Cy3 filters (excitation: 531/40 nm and emission: 593/40 nm). Nuclear count and percentage of cells staining positive for propidium iodide were analysed using the multi-wavelength cell scoring module on the MetaXpress 6 software (Molecular Devices). To confirm apoptotic cell death, protein was isolated 24 h after cells were treated with apoptosis inducers (navitoclax in MDA-MB-231 cells or S63845 (A8737, ApexBio Technology, Houston, TX, USA) in MDA-MB-468 and HCC1806 cells).

### Quantitative real-time Polymerase Chain Reaction (qPCR)

RNA was isolated using the Qiagen RNeasy Mini Kit (Qiagen, Venlo, Netherlands) and reverse transcribed using the Omniscript RT kit (Qiagen). cDNA was amplified using the TaqMan Fast Universal PCR Master Mix (Applied Biosystems, Thermo Fisher Scientific). Real-time PCR was performed using the StepOne Plus Real-Time PCR System (Applied Biosystems). Taqman gene expression assays used were TMCO1 (Hs00976965_m1), SMAD7 (Hs00998193_m1) and PGK1 (Hs99999906_m1). PGK1 was used as the housekeeping gene.

### Immunoprecipitation and liquid chromatography mass spectrometry

Parental MDA-MB-231 cells (control) and MDA-MB-231 cells stably overexpressing FLAG-TMCO1 were used for affinity purification and subsequent MS analysis as previously described [68]. Briefly, cells were lysed in TAP buffer (10% glycerol, 50 mM HEPES-NaOH (pH 8.0), 150 mM NaCl, 2 mM EDTA, 0.1% NP-40, 2 mM DTT, 1X protease inhibitor, 1X phosphatase inhibitor). Cleared protein lysate was incubated with pre-washed anti-FLAG-M2 beads (A2220, Sigma-Aldrich) and incubated at 4 °C on a rotator for 16 h. Beads were then washed 3 times with TAP buffer, 3 times with 50 mM Ammonium bicarbonate and resuspended in 50 µL of 50 mM ammonium bicarbonate. On-beads trypsin digestion was performed overnight and peptides were dried by speed-vac. Samples were resuspended and desalted with C18 tip (Pierce™ C18 Spin Tips, #84850, ThermoFisher Scientific) according to the manufacturers’ protocol and dried by speed-vac. Samples were resuspended in 26 µl of 1% (vol/vol) formic acid, and centrifuged at 13,200 RPM for 20 min. 10 µl of digested peptides was used for analysis by liquid chromatography-tandem mass spectrometry (LC-MS/MS) on a Thermo Q-Exactive HF quadrupole-Orbitrap mass spectrometer (Thermo Fisher Scientific). Acquired spectra were searched against a FASTA file containing the human NCBI sequenced on the Sorcerer platform. Three biological replicates of control and FLAG-TMCO1 IPs were performed.

### Subcellular fractionation and assessment of nuclear translocation

Cell fractionation into cytosolic, membrane/organellar and nuclear-enriched subcellular fractions were performed using the Subcellular Protein Fractionation Kit for Cultured Cells (Thermo Fisher Scientific) according to the manufacturer’s protocol. Subcellular fractions were obtained using cell densities of approximately 1.5–2 × 10^6^ cells and were harvested from MDA-MB-231 cells seeded in T25 flasks. To assess SMAD2 translocation, cells were washed once with serum-free media and incubated with either vehicle control (0.1% BSA) or TGFβ (10 ng/mL) for 1 h in serum-free media prior to cell fractionation. Cell fractionation experiments involving TMCO1 siRNA transfections were done in 96-well plates using optimised volumes of fractionation buffers from the fractionation kit.

### Quantification and statistical analysis

All data are presented as mean ± S.D. Data were organised in Microsoft Excel and graphed and analysed using GraphPad Prism (ver. 9.5.1, GraphPad Software). At least three biological replicates were used for experiments. Statistical tests used and corresponding *p*-values are detailed in the figure legends. *P*-values < 0.05 were considered significant. Any normalised data shown are relative to control treatments at the start of treatment, if applicable.

## Supplementary information

Supplementary information and uncropped western blots

Table S2

## Data Availability

Relevant data related to this manuscript will be available upon request for non-commercial uses. Mass spectrometry data generated in this study is publicly available.
