# Supplementary material for: TMCO1 is upregulated in breast cancer and regulates the response to pro-apoptotic agents in breast cancer cells
Source: Cell Death Discov. 2024 Oct 1;10:421. doi: 10.1038/s41420-024-02183-0 (PMC11445413; doi:10.1038/s41420-024-02183-0)

Supplementary Information

TMCO1 is upregulated in breast cancer and regulates the response to pro-apoptotic agents in breast cancer cells

Alice HL Bong[1], Mélanie Robitaille[1], Sichun Lin[2], Amy McCart-Reed[3], Michael Milevskiy[4,5], Stéphane Angers[2,6,7], Sarah J Roberts-Thomson[1], Gregory R Monteith[1].

1. School of Pharmacy, The University of Queensland, Woolloongabba, QLD Australia.
2. Donelly Centre, University of Toronto, Toronto, ON M5S 1A8, Canada
3. UQ Centre for Clinical Research, Faculty of Medicine, The University of Queensland, Herston, QLD, Australia
4. ACRF Cancer Biology and Stem Cells, The Walter and Eliza Hall Institute of Medical Research, Parkville, Victoria.
5. Department of Medical Biology, The University of Melbourne, Parkville, Australia.
6. Leslie Dan Faculty of Pharmacy, University of Toronto, Toronto, ON M5S 1A2, Canada.
7. Department of Biochemistry, Temerty Faculty of Medicine, University of Toronto, Toronto, ON M5S 1A8, Canada.

Supplementary Tables

Table S1: Table shows the survival analysis of breast cancer patients with high or low *TMCO1* expression based on their PAM50 molecular subtypes and lymph node involvement using Kaplan-Meier Plotter.

PAM50 subtype	Lymph node status	Logrank P	Hazards ratio (HR)	95% CI	Patient numbers (*n*)	
					Low *TMCO1*	High *TMCO1*
Luminal A	Negative	0.1825	1.24	0.9 – 1.7	359	630
	Positive	0.3792	0.84	0.57 – 1.24	198	333
Luminal B	Negative	0.4008	1.13	0.85 – 1.48	264	346
	Positive	0.0732	1.34	0.97 – 1.83	190	291
HER2	Negative	0.231	0.74	0.45 – 1.22	163	101
	Positive	0.3366	0.84	0.58 – 1.21	94	183
Basal	Negative	0.1118	0.73	0.49 – 1.08	324	132
	Positive	0.0063	1.68	1.15 – 2.44	233	90

Table S2: Table showing number of total and unique peptides as well as peptide coverage for proteins identified to interact with FLAG-TMCO1 in LC-MS/MS.

(separate Excel file)

Supplementary Figures

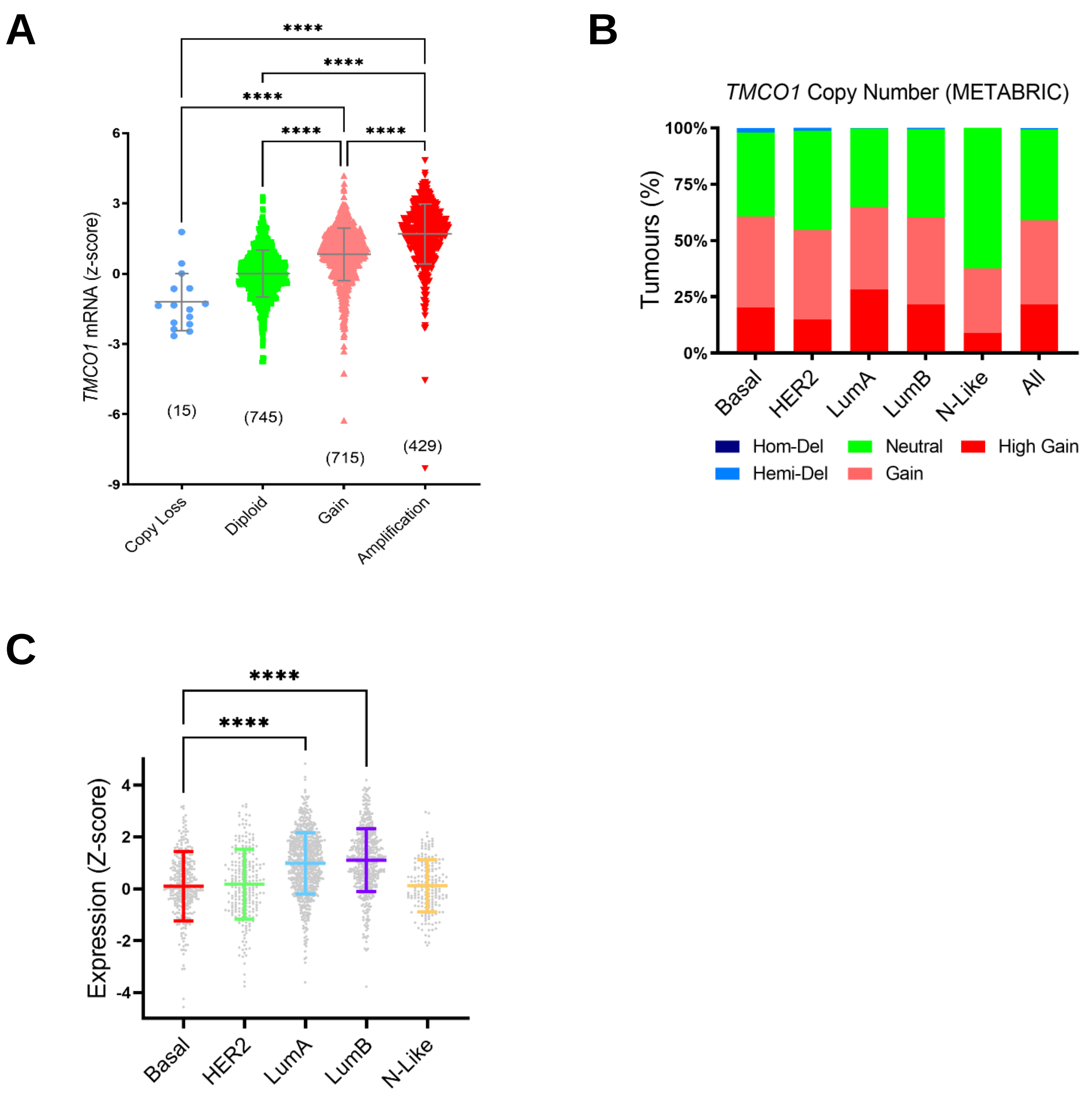

Supplementary Fig. 1: *TMCO1* gene copy number and gene expression in the METABRIC breast cancer patient dataset

A *TMCO1* mRNA levels in METABRIC breast cancer patient dataset stratified to gene copy number status. Sample sizes for individual gene copy number groups are shown in the brackets. ****$P < 0.0001$ (one way ANOVA, Kruskal-Wallis test) **B** Proportion of *TMCO1* copy number alterations within the different breast cancer molecular subtypes in the METABRIC dataset **C** *TMCO1* gene expression classified to breast cancer molecular subtypes. (one way ANOVA, Tukey's test) Error bars represent mean ± S.D.

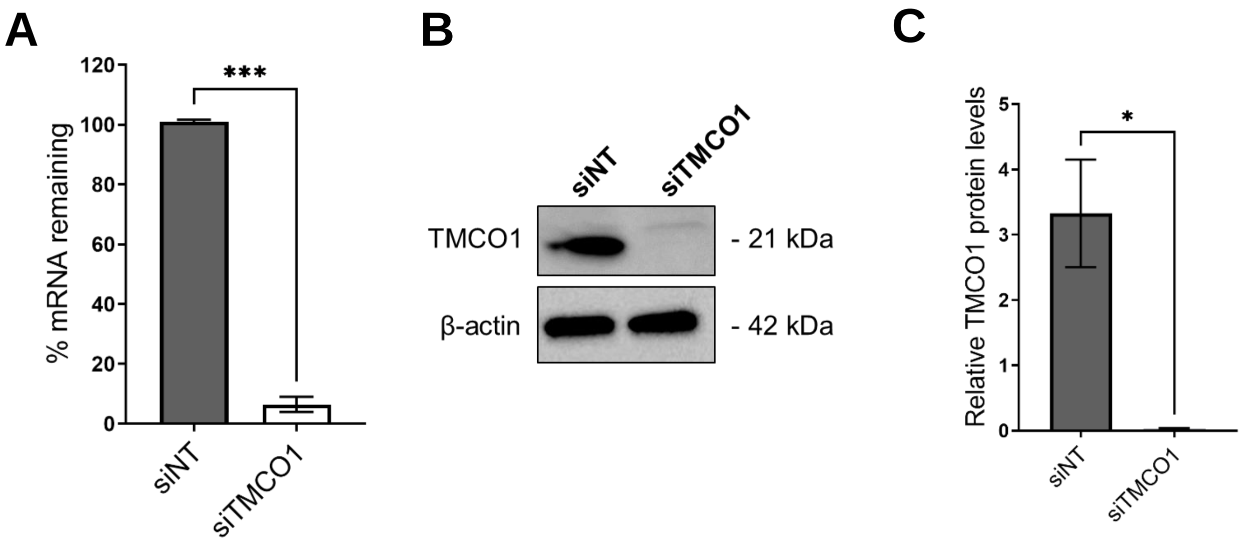

Supplementary Fig. 2: Validation of *TMCO1* silencing using Dharmacon ON-TARGETplus TMCO1 siRNA

A Bar graph shows percentage of *TMCO1* mRNA remaining in GCaMP6m-MDA-MB-231 cells at 48 – 72 h following transfection with Dharmacon ON-TARGETplus TMCO1 siRNA. **B, C** Representative immunoblot and densitometric analysis showing TMCO1 protein silencing at 96 h post-siRNA transfection. * $P < 0.05$, **** $P < 0.0001$ (paired t-test). Bar graphs show mean ± S.D.

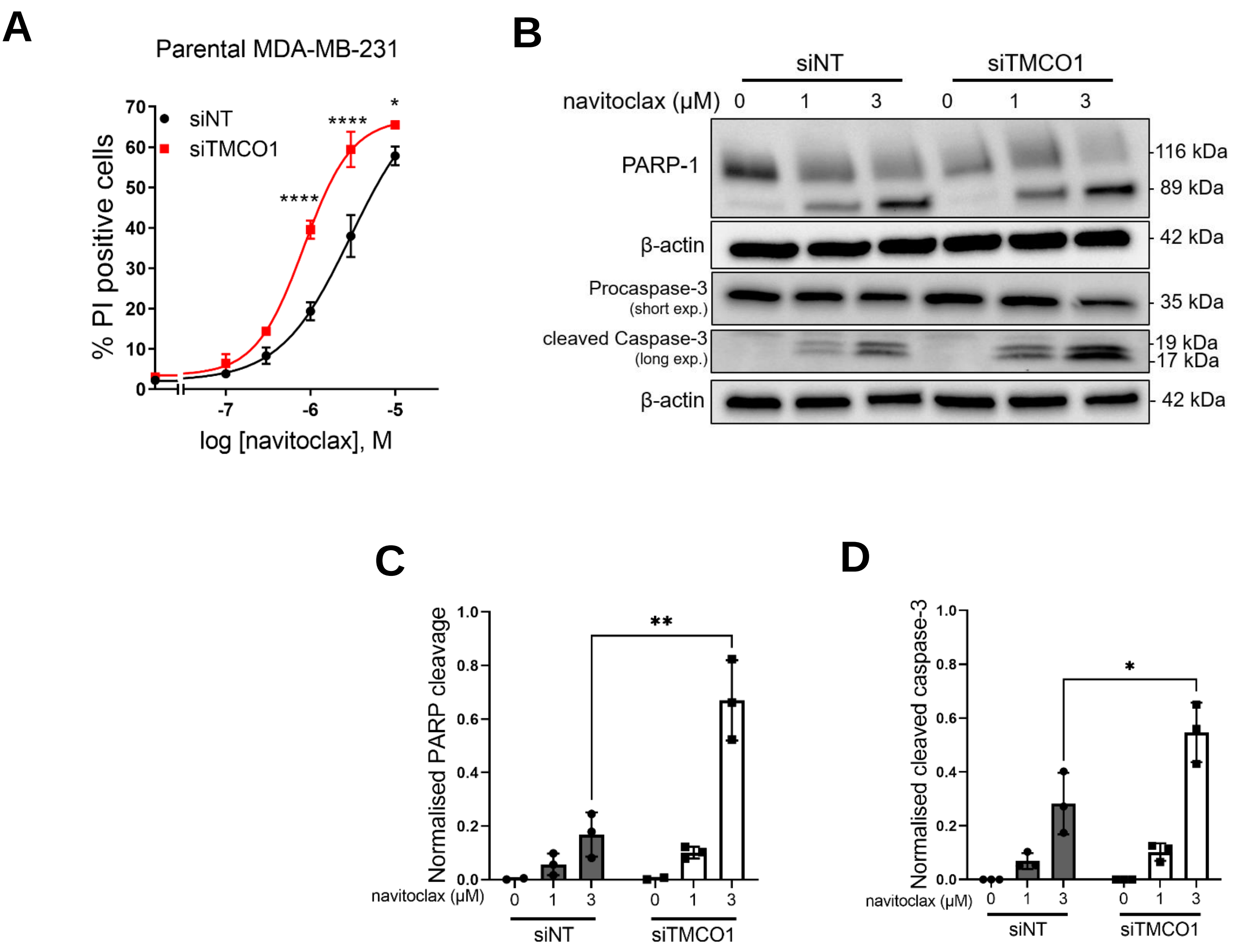

Supplementary Fig 3: Effect of *TMCO1* silencing on cell death in parental MDA-MB-231 cells

A Concentration-effect curve showing %PI positivity at different concentrations of navitoclax. Data points represent mean ± S.E.M (n = 3) **B** Representative immunoblots showing increased PARP-1 and caspase-3 cleavage as a result of *TMCO1* silencing in parental MDA-MB-231 cells treated with navitoclax. **C, D** Densitometric analyses showing the effect of *TMCO1* silencing on PARP-1 and caspase-3 cleavage with navitoclax treatment. Bar graphs show mean ± S.D. (n = 3) * $P < 0.05$, ** $P < 0.01$, **** $P < 0.0001$ (two-way ANOVA with Sidak's post-hoc test).

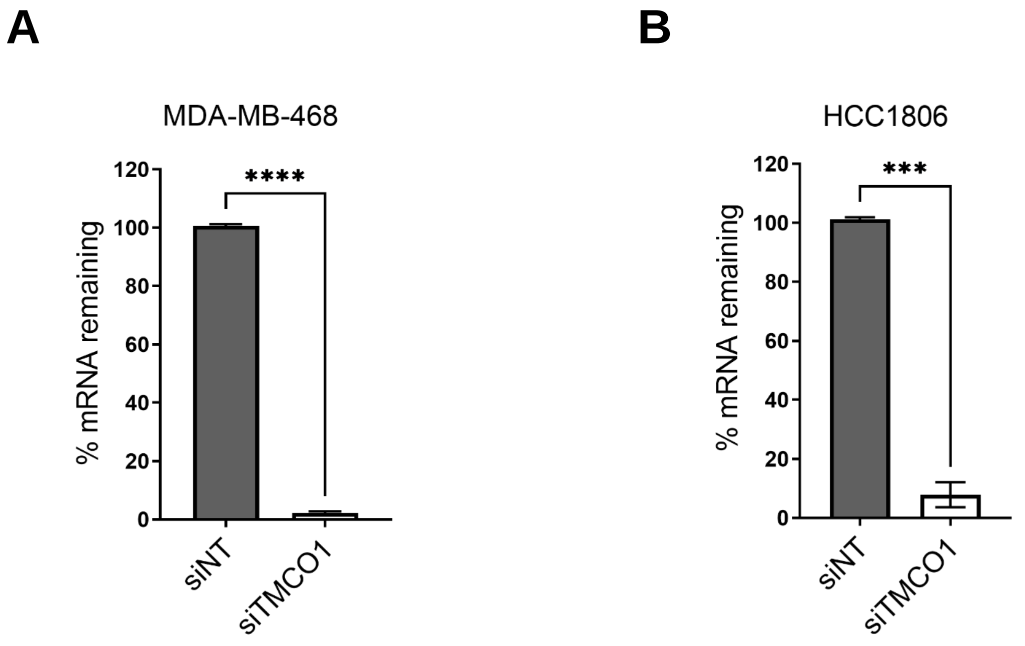

Supplementary Fig 4: Validation of *TMCO1* silencing in MDA-MB-468 and HCC1806 cell lines

A, B Bar graphs show percentage of *TMCO1* mRNA remaining in MDA-MB-468 and HCC1806 cells at 48 h following transfection with Dharmacon ON-TARGETplus TMCO1 siRNA. **** $P < 0.0001$ (paired t-test). Bar graphs show mean ± S.D.

Full-length Western blots

Figure 4A

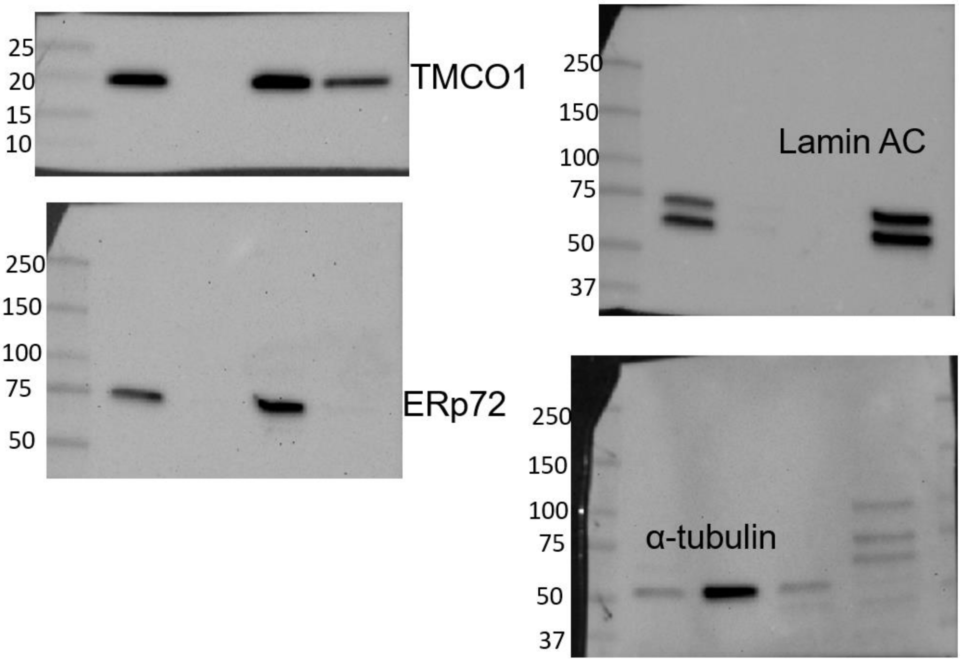

Figure 4F

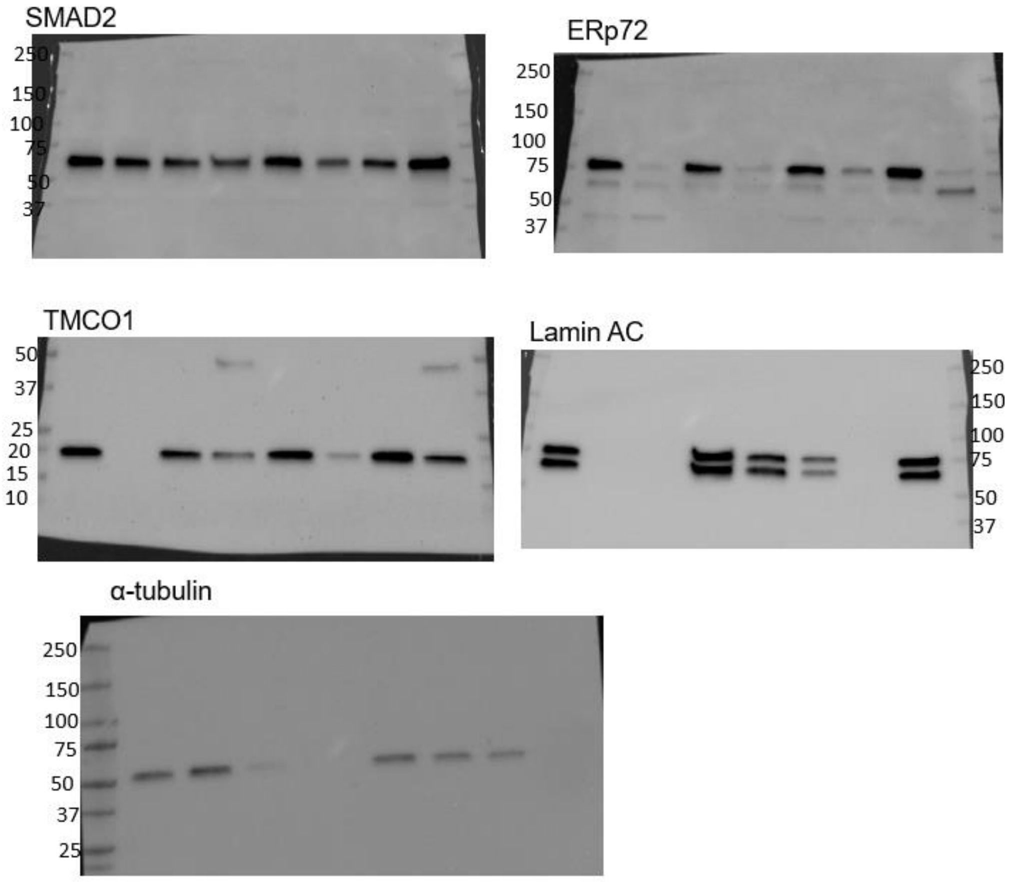

Figure 4I

SMAD2

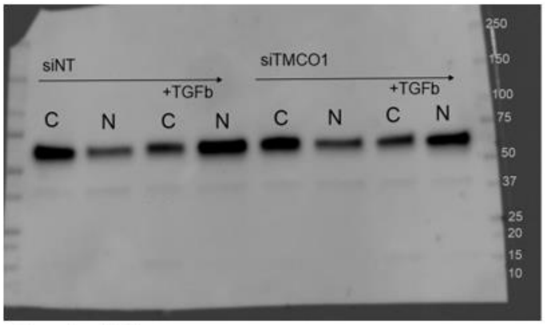

Lamin A/C

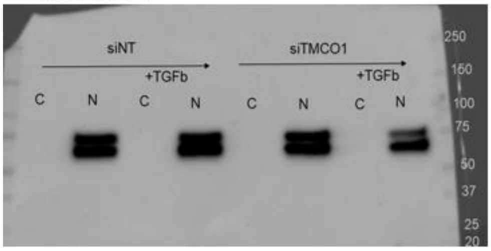

α-tubulin

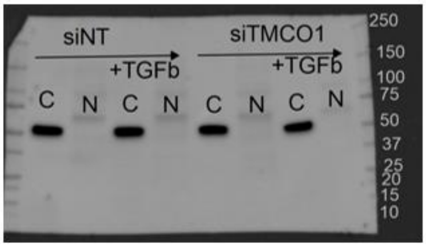

Figure 5C

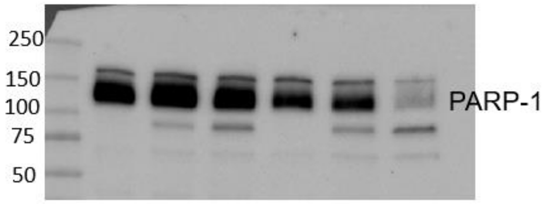

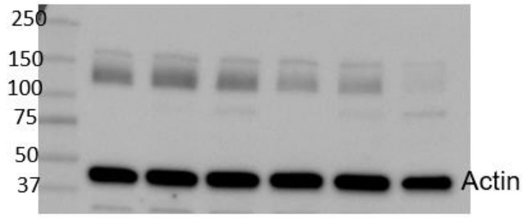

Caspase 3 (short exposure)

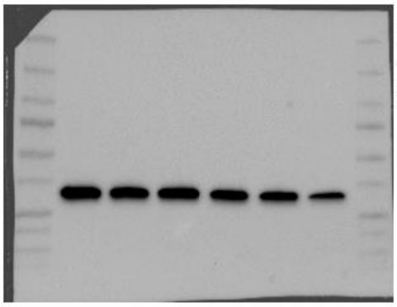

Caspase 3 (long exposure)

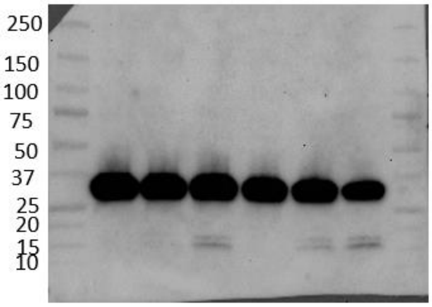

Actin for caspase 3

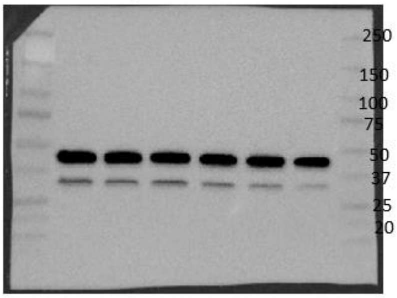

Figure 5F

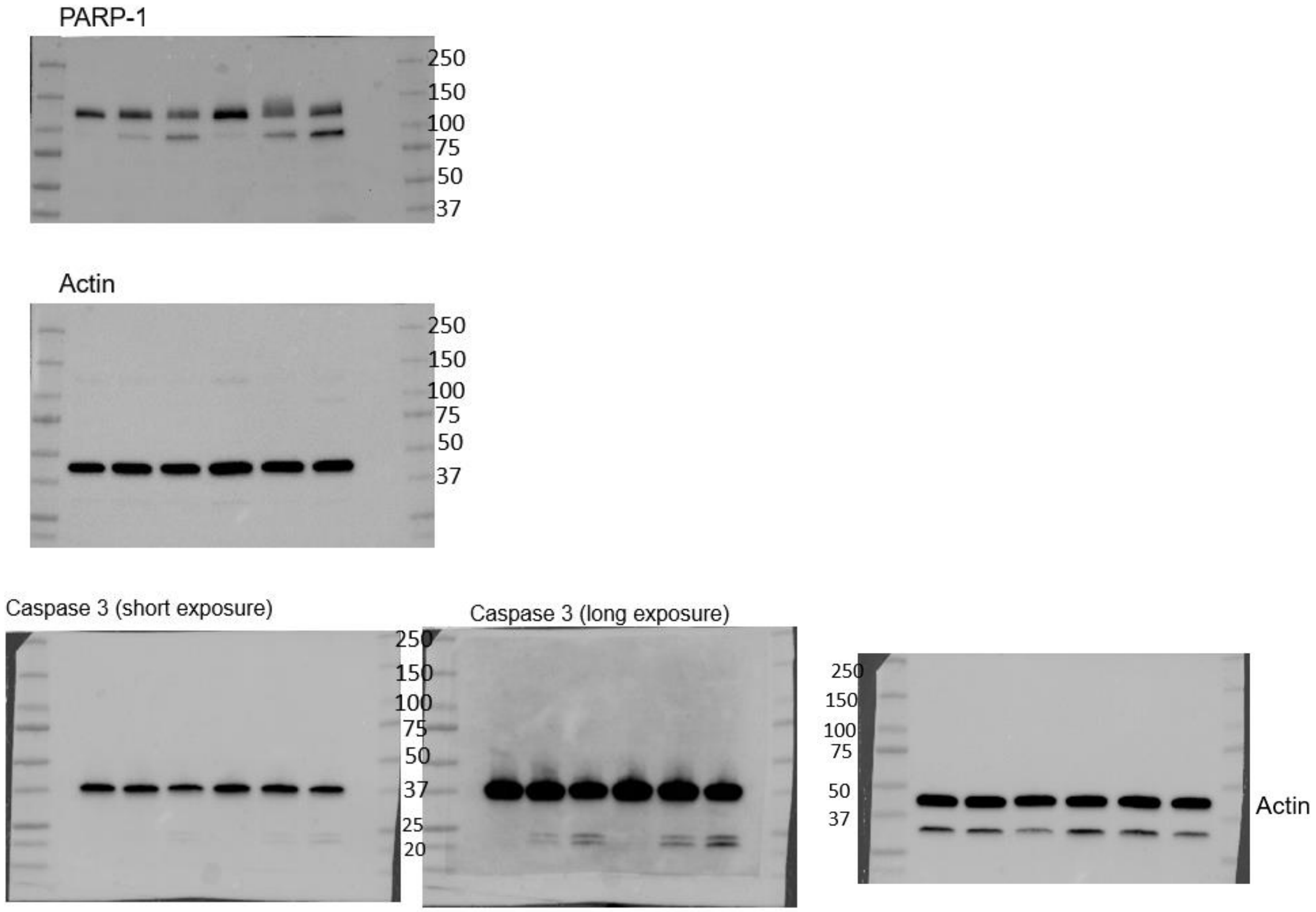

Figure 6C

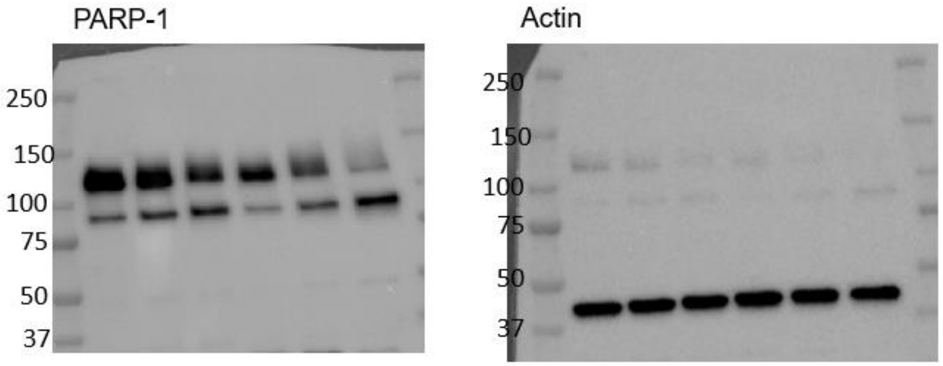

Figure 6E

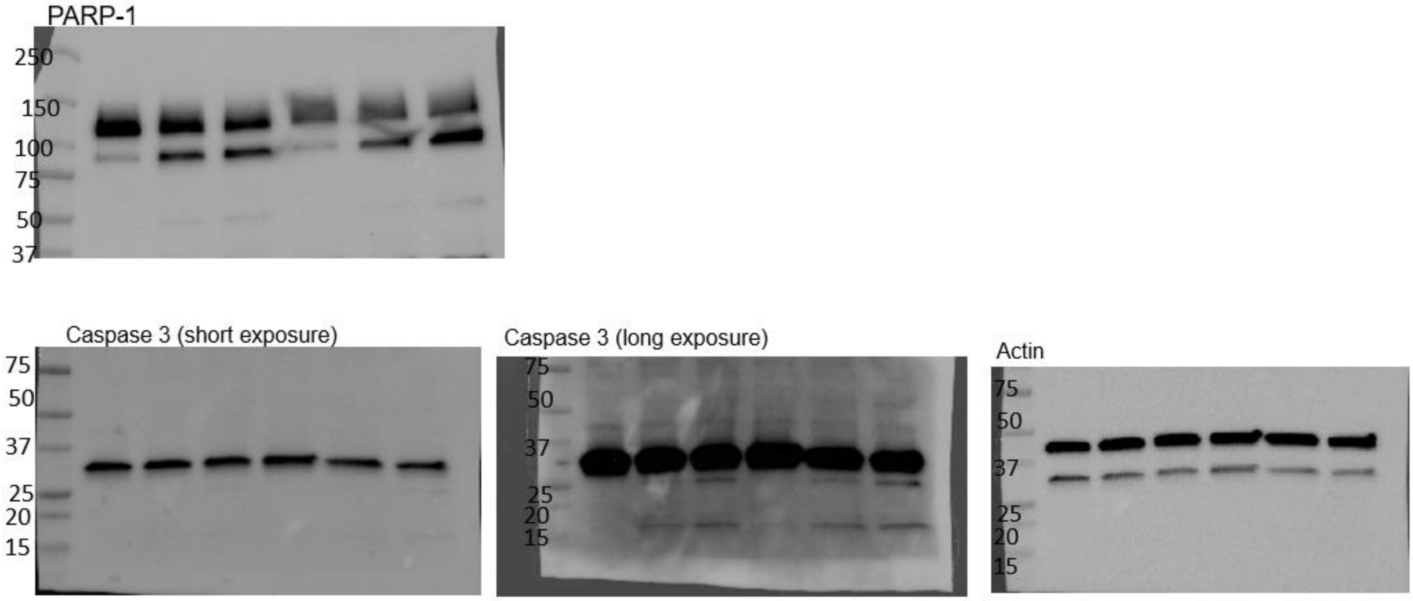

Supp Fig 2

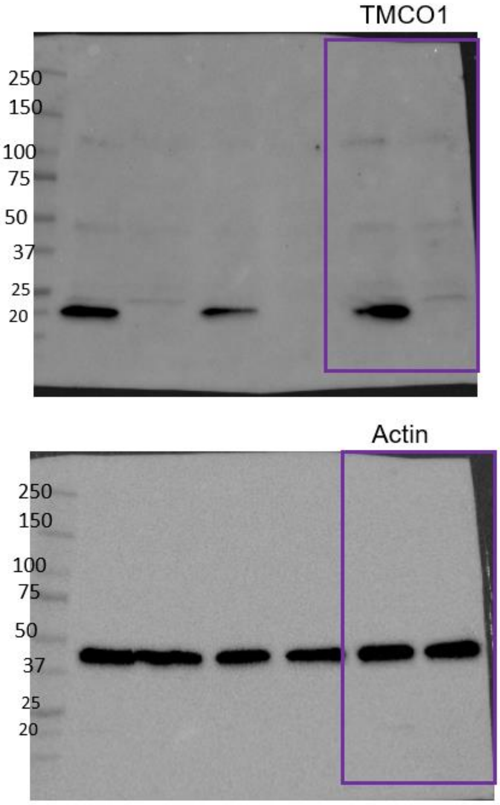

Supp Fig 3

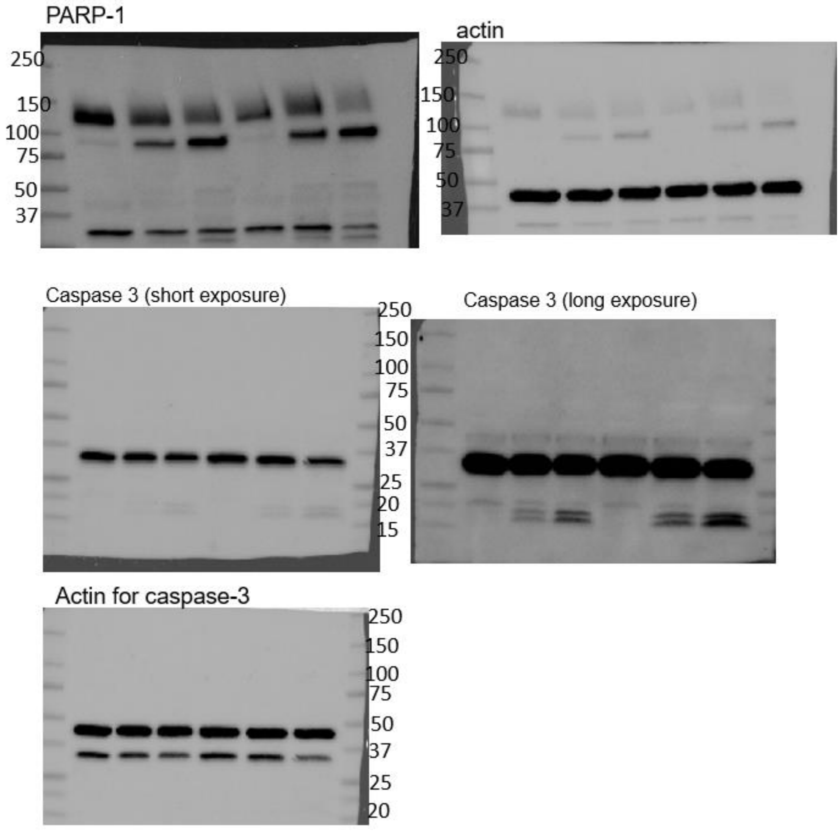

Supplement: Supplementary file 1 — Supplementary information and uncropped western blots [file 41420_2024_2183_MOESM1_ESM.pdf]
